# Introducing extracorporeal membrane oxygenation (ECMO) for healthcare professionals: the importance of a basic theoretical course

**DOI:** 10.1186/1749-8090-8-S1-P52

**Published:** 2013-09-11

**Authors:** G Liguori, J Penha, L Miana, L Caneo, C Tanamati, M Jatene

**Affiliations:** 1 Heart Institute (InCor), University of Sao Paulo Medical School, Sao Paulo, Brazil

## Introduction

A multidisciplinary team is essential for a high quality ECMO service. However, few health professionals have any knowledge on ECMO and, in addition, there are discrepancies between the level of expertise among these professionals. Thus, we evaluated the impact of a basic theoretical ECMO course on the knowledge gain of different healthcare professionals.

## Methods

A basic six-hour theoretical course on ECMO was taught to a heterogeneous audience formed by 12 medical students, 10 nurses, 4 perfusionists and 4 physicians. Content included the following topics: 1) concept of ECMO; 2) usage scenarios; 3) aspects of the circuit; 4) differences between pulmonary and cardiac ECMO; 5) management of patients on ECMO; 6) aspects of cannulation; 7) complications; 8) concept of E-CPR; and 9) importance of the multidisciplinary team. Questionnaires for self-evaluation, graduating from 1 to 5 the level of expertise on the referred topics, were conducted immediately before and after the course.

## Results

Twenty-six (76,6%) questionnaires were completed. The three most clear topics before the course were the importance of the multidisciplinary team (2,96±1,36), the concept of ECMO (2,57±1,27) and usage scenarios (2,39±1,2), while the three less clear were the concept of E-CPR (1,65±0,93), management of patients on ECMO (1,87±1,01) and aspects of cannulation (1,91±1,12). An important, although not significant, discrepancy between the level of expertise among the professional categories was present before the course (p=0,157). After the course, a significant knowledge gain was observed in all the topics (p<0.001). Perfusionists presented lower gain than others participants (p=0.05). At the end, there was an homogenization between the level of expertise among the audience (p<0,001).

## Conclusion

A basic theoretical ECMO course is important, not only to raise the expertise on ECMO, but also to level different professionals for future advanced training and specialization.

